# A scoping review of factors associated with pain empathy among nursing professionals and students

**DOI:** 10.3389/fpsyg.2026.1916376

**Published:** 2026-09-07

**Authors:** Jingjing Li, Lihua Wu, Dandan Gao, Yang Gao

**Affiliations:** 1 Shanxi University of Chinese Medicine, Jinzhong, China; 2 Shanxi Bethune Hospital, Taiyuan, China

**Keywords:** empathy, nursing, nursing students, pain, scoping review

## Abstract

**Objective:**

To map the factors associated with pain empathy among nursing professionals and students and provide a theoretical basis for intervention research.

**Methods:**

Guided by the methodological framework of scoping review, databases including PubMed, Embase, Web of Science, Cochrane Library, CNKI, VIP, Wanfang Data Knowledge Service Platform, and Chinese Biomedical Literature Database were searched for studies on factors associated with pain empathy. The search period was from the establishment of each database to December 16, 2025. Two researchers independently screened literature and extracted data, and the included studies were summarized and analyzed.

**Results:**

A total of 27 studies were included: 23 cross-sectional, 3 qualitative, and 1 quasi-experimental. Sixteen used the Empathy for Pain Scale, 7 used general-empathy measures in pain-related contexts, 1 used fMRI, and 3 were qualitative. The reported factors included personal background, work experience, training history, pain-related factors, psychological and emotional state, and social support and environment.

**Conclusion:**

Pain empathy among nursing professionals and students was associated with multiple factors, but the heterogeneous constructs and predominantly cross-sectional evidence do not support causal inference. Future longitudinal and intervention studies are needed.

## 1 Introduction

Pain is defined as an unpleasant sensory and emotional experience associated with actual or potential tissue damage, recognized as a subjective perceptual phenomenon (Raja et al., 2020). The World Health Organization has classified pain as the fifth vital sign, highlighting its evolution into a critical global public health challenge. Statistical data indicate that over 300 million people in China suffer from chronic pain (Yongjun et al., 2020), which ranks third in morbidity after cardiovascular diseases and malignancies. Pain exerts multi-dimensional and long-lasting adverse impacts on patients. Physiologically, it triggers autonomic nervous system disorders, immunosuppression, and sleep disturbances; psychologically, it increases the risk of generalized anxiety, depression, and even suicidal ideation (Scarlata and Mancini, 2023). From a social perspective, persistent pain contributes to disability, increased care dependency, and heavy economic burdens, further impairing patients’ quality of life, reducing treatment adherence, and prolonging rehabilitation cycles (Zhang and Shen, 2022).

As frontline practitioners in pain management, registered nurses play an indispensable core role in pain assessment, intervention, and symptom alleviation (Allen et al., 2018). Pain management requires standardized technical operations as well as humanistic nursing grounded in empathic care (Roche and Harmon, 2017). Given the multi-dimensional subjectivity of pain perception, clinical nurses are required to possess proficient empathic capacity to accurately identify patients’ painful conditions. Pain empathy is a specialized subtype of general empathy, describing the psychological and neurological process whereby individuals perceive, understand, and vicariously share others’ painful feelings (Jackson et al., 2005). Existing evidence demonstrates that nurses with superior pain empathy tend to actively listen to patients’ complaints, lower the risk of pain underestimation, and integrate both physical and psychological demands into clinical decision-making (Keumseong et al., 2022). Despite the progressive popularization of humanistic nursing philosophy in recent years, cross-sectional investigations reveal that Chinese nurses’ overall pain empathy remains at a moderate level (Wu et al., 2022; Gao et al., 2022). High-level pain empathy enables nurses to collect comprehensive clinical information related to patients’ pain, boost patients’ participation in therapeutic care (Moudatsou et al., 2020), enhance the precision of pain evaluation, and alleviate patients’ psychological dread toward pain (Nembhard et al., 2023). Accordingly, clarifying contributing factors of nurses’ pain empathy bears important theoretical and practical significance for optimizing pain nursing quality and improving patient-reported pain outcomes.

As an evidence synthesis approach rooted in evidence-based practice, scoping review facilitates researchers to map research progress, classify existing evidence sources, summarize research achievements, and identify unresolved research gaps efficiently (Davis et al., 2009). Following the scoping review framework proposed by Arksey and O’Malley (Arksey and O'malley, 2005) and PRISMA-ScR reporting guidance (Tricco et al., 2018), this study maps available literature on factors associated with pain empathy and identifies gaps for subsequent research.

## 2 Materials and methods

### 2.1 Formulation of research questions

Using the PCC framework (Peters et al., 2020), the review question was: What factors associated with pain empathy have been reported among nursing professionals and students in nursing education and clinical practice? Participants included clinical nurses, nursing interns, nursing assistants, and nursing students. The concept was pain empathy and its associated factors, measured with pain-specific instruments or general-empathy instruments used in pain-related contexts. The context comprised nursing education and clinical practice in the countries represented by the included evidence.

### 2.2 Literature search strategy

Eight electronic databases, including PubMed, Embase, Web of Science, Cochrane Library, CNKI, VIP, Wanfang Data, and CBM, were searched from their respective dates of establishment to December 16, 2025. Database-specific yields were PubMed 171, Embase 289, Web of Science 644, Cochrane Library 184, CNKI 33, VIP 21, Wanfang Data 1,388, and CBM 10, totaling 2,740 records. Subject headings and free-text terms for pain empathy and nursing populations were combined. The exact database-specific syntax was transcribed from the saved search screenshots and reproduced in Supplementary Table S1. Before resubmission, a second reviewer independently checked the database adaptations, Boolean and proximity logic, subject headings, free-text terms, limits, spelling, and syntax against the PRESS 2015 elements; the review summary is provided in Supplementary Table S7.

### 2.3 Inclusion and exclusion criteria

Inclusion Criteria: (1) The study population comprised clinical nurses, nursing interns, nursing assistants, and nursing students; (2) The study examined pain empathy, or general empathy in an explicitly pain-related context, and reported associated factors or intervention-related changes; (3) Original quantitative, qualitative, mixed-method, or quasi-experimental primary studies; (4) Publications written in Chinese or English.

Exclusion Criteria: (1) Duplicate publications; (2) Conference abstracts, review articles, and other non-original studies; (3) Studies measuring only general empathy without a pain-related context. Reports that could not be retrieved were recorded separately and were not counted as full-text exclusions.

### 2.4 Literature screening and data extraction

EndNote software was utilized for document management. Two researchers independently screened titles and abstracts and then completed the report-level retrieval/eligibility disposition. Disagreements were resolved through discussion or consultation with a third researcher. Cohen’s kappa was 0.82 for title/abstract screening. For the next stage, the agreement table retained all 87 reports sought for retrieval: the 5 reports that could not be retrieved were coded as a separate negative disposition and were not counted among the 55 full-text exclusions; kappa was 0.79 for this 87-report retrieval/eligibility disposition. Two researchers also checked extraction-table completeness independently (kappa = 0.85). Extracted fields included first author, publication year, country, research design, sample size, measurement instrument or data source, participant group, construct class, reported factor, effect direction and metric, and independent-cohort identifier.

### 2.5 Methodological quality appraisal

Two reviewers independently applied the design-appropriate JBI critical appraisal checklist: the 8-item analytical cross-sectional checklist, 10-item qualitative checklist, or 9-item quasi-experimental checklist. Items were recorded as Yes, No, Unclear, or Not applicable, with disagreements resolved by discussion. Item-level consensus results were used to calibrate interpretation and were not converted into a composite quality score or used as an exclusion criterion. The checklist type and consensus item matrix are reported in Supplementary Table S3.

### 2.6 Data synthesis and construct stratification

Evidence was summarized descriptively by construct, factor, design, participant group, and independent cohort. Scores from different instruments were not pooled. Cross-sectional findings are described as associations; intervention-related change is reported separately for the quasi-experimental study.

### 2.7 Protocol and reporting

The review protocol was not prospectively registered, and grey literature was not searched. Reporting was checked against PRISMA-ScR domains. These limitations are stated in the manuscript.

## 3 Results

### 3.1 Literature screening outcomes

**Figure 1:**
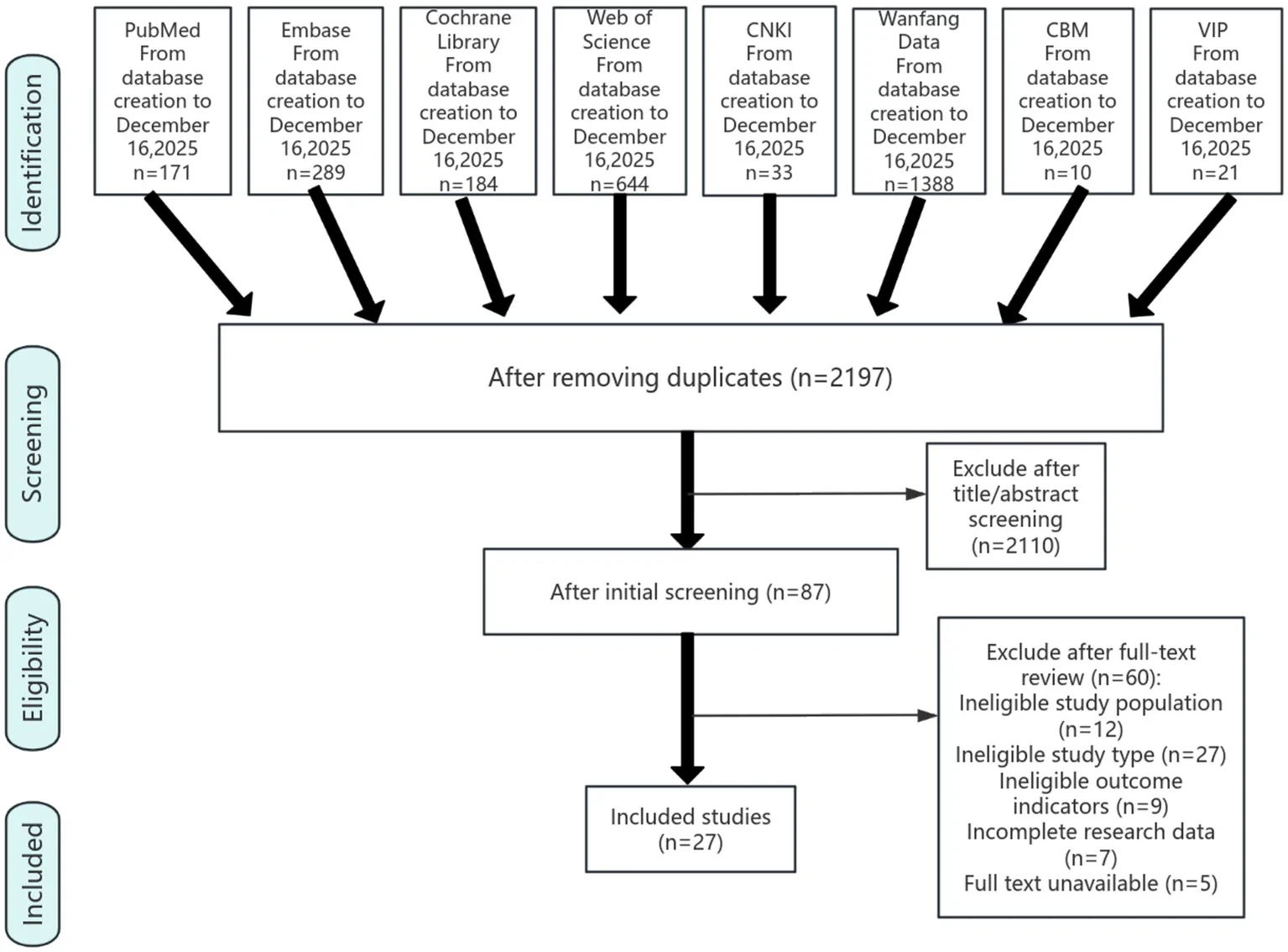
PRISMA-ScR literature screening flowchart.

The database searches identified 2,740 records. After 543 duplicates were removed, 2,197 records were screened by title and abstract, of which 2,110 were excluded. Eighty-seven reports were sought for retrieval; five were not retrieved. Eighty-two full-text reports were assessed, and 55 were excluded with reasons, leaving 27 studies for inclusion. Full-text exclusions are listed in Supplementary Table S2, reports not retrieved in Supplementary Table S2a, and the PRISMA-ScR flow is shown in Figure 1.

### 3.2 Basic characteristics of included literature

The 27 included articles were published between 2014 and 2025, comprising 12 Chinese papers and 15 English papers. Geographically, 16 studies were conducted in mainland China, 1 in Taiwan, and 10 in France, the United Kingdom, the United States, Canada, or Turkey. Detailed baseline information is listed in Table 1.

**Table 1:** Basic characteristics of the included literature (*n* = 27).

First author	Year	Country	Study participants	Study type	Sample size	Instrument/data source	Reported associated factors
Dong et al. (2024)	2024	China	Clinical nurses	Cross-sectional study	953	a	Resilience, gender, marital status, department, religious belief, pain empathy-related training, experience of severe traumatic pain
Gao et al. (2022)	2022	China	Clinical nurses	Cross-sectional study	687	a	Professional title, time spent on pain assessment, pain resource nurse status, total weekly working hours, emotional empathy
Ren et al. (2023)	2023	China	Clinical nurses	Cross-sectional study	9,168	a	Age, professional title, highest educational background, pain knowledge training, knowledge sharing behavior
Luo et al. (2024)	2024	China	ICU nurses	Cross-sectional study	324	a	Self-efficacy in pain management
Sun et al. (2022)	2022	China	Pediatric nurses	Cross-sectional study	132	a	Pediatric working years, childbearing status, pain empathy-related training, workplace violence experience, professional quality of life
Wu et al. (2022)	2022	China	Clinical nurses	Cross-sectional study	489	a	Traumatic/pain experience, negative emotions, perceived pain tolerance, participation in pain quality control, empathy concept awareness, job satisfaction, oncology nursing experience, personality traits
Zhang and Kuang (2024)	2024	China	Clinical nurses	Cross-sectional study	254	a	Professional title, membership in department pain management group, childbearing status, emotional empathy
Zhang et al. (2025)	2025	China	ICU nurses	Cross-sectional study	394	a	Perceived social support
Shen et al. (2024)	2024	China	Infectious diseases nurses	Cross-sectional study	271	a	Religious belief, experience of severe traumatic pain, pain management training
Li et al. (2024)	2024	China	Nursing students	Cross-sectional study	230	a	Gender, educational background, only-child status, preference for violent movies, parental rearing style
Xiang et al. (2023)	2023	China	Nursing students	Cross-sectional study	653	ab	Exposure to violent/bloody videos, pain-related experience, perceived pain tolerance, pain empathy awareness, cognitive and emotional empathy
Kuo et al. (2022)	2022	Taiwan, China	Urology nurses	Qualitative research	9	Interview outline	Reading and narrative intervention
Huang et al. (2024)	2024	China	Clinical nurses	Cross-sectional study	1,601	a	Resilience, coping style
Wu et al. (2023)	2023	China	Clinical nurses	Cross-sectional study	177	a	Pain knowledge, empathy training, personal pain experience, emotional state
Li Q. et al. (2025)	2025	China	Nursing students	Cross-sectional study	423	a	Humanistic care education, operating room internship experience, pain experience, interpersonal relationships during internship, family rearing mode, meaning in life
Li B. et al. (2025)	2025	China	Clinical nurses	Cross-sectional study	1,601	a	Perceived pain tolerance, job satisfaction, coping style, age, marital status, childbearing status, pain-related training, pain resource nurse status
Huang et al. (2025)	2025	China	Clinical nurses	Cross-sectional study	1,601	a	Resilience level, educational level, only-child status, personality traits, workplace violence experience, job satisfaction
Bourgault et al. (2015)	2015	France	Emergency nurses	Cross-sectional study	30	c	Psychological well-being
Mankelow et al. (2020)	2020	United Kingdom	Nursing students	Qualitative research	12 (including 2 nursing students)	Interview outline	Pain neurophysiology education (PNE) lectures
Paul-Savoie et al. (2018)	2018	Canada	Clinical nurses	Cross-sectional study	21	cf	Pain visibility in patients with chronic pain
Latimer et al. (2017)	2017	Canada	Neonatal and pediatric intensive care nurses	Cross-sectional study	27	b	Occupational characteristics
Jackson et al. (2017)	2017	Canada	Neonatal and pediatric intensive care nurses	Cross-sectional study	27	e	Occupational characteristics
Simko et al. (2021)	2021	United States	Nursing students	quasi-experimental study	182	cd	Chronic pain simulation experience
Dobbs et al. (2014)	2014	United States	Nurse auxiliaries	Qualitative research	28	Interview outline	Personal pain experience
Özbaş (2025)	2025	Turkey	Nurses in surgical clinics, operating rooms, and intensive care units	Cross-sectional study	242	g	Age, educational level, working years, work shift, job satisfaction, pain and empathy-related training, personal surgical experience, acute pain experience, pain knowledge and attitudes
Yurdagül (2025)	2025	Turkey	Nursing students	Cross-sectional study	396	h	Fear of pain
Culha (2025)	2025	Turkey	Nursing students	Cross-sectional study	326	i	Emotional intelligence, academic grade, chronic pain experience, self-efficacy in pain management

a. Empathy for Pain Scale (EPS), b. Interpersonal Reactivity Index (IRI), c. Jefferson Scale of Empathy (JSE), d. Kiersma-Chen Empathy Scale (KCES), e. Functional Magnetic Resonance Imaging (fMRI), f. Reynolds Empathy Scale (RES), g. Basic Empathy Scale (BES), h. Empathic Tendency Scale (ETS), i. Multidimensional Emotional Empathy Scale.

### 3.3 Study design and construct classification

The 27 studies comprised 23 cross-sectional, 3 qualitative, and 1 quasi-experimental study. Design-appropriate item-level JBI results are presented in Supplementary Table S3; no composite quality score was calculated.

### 3.4 Current status of nurses’ pain empathy

The included studies used heterogeneous outcome approaches: 16 used the Empathy for Pain Scale (EPS), 7 used general-empathy instruments in pain-related contexts, 1 used fMRI, and 3 were qualitative (Wu et al., 2022; Gao et al., 2022; Dong et al., 2024; Ren et al., 2023; Luo et al., 2024; Sun et al., 2022; Zhang and Kuang, 2024; Zhang et al., 2025; Shen et al., 2024; Li et al., 2024; Li Q. et al., 2025; Li B. et al., 2025; Xiang et al., 2023; Kuo et al., 2022; Huang et al., 2024; Wu et al., 2023; Huang et al., 2025; Bourgault et al., 2015; Mankelow et al., 2020; Paul-Savoie et al., 2018; Latimer et al., 2017; Jackson et al., 2017; Simko et al., 2021; Dobbs et al., 2014; Özbaş, 2025; Yurdagül, 2025; Culha, 2025). EPS studies reported moderate or high scores as described in the individual reports (Ren et al., 2023; Li Q. et al., 2025). General-empathy and qualitative findings were not converted to EPS levels, and the fMRI study was retained as a neural correlate without high/medium/low classification. Construct-stratified results are shown in Supplementary Table S4. In an EPS-restricted descriptive analysis, the reported directions for education/training, resilience, pain knowledge, job satisfaction, and personal pain experience were compared with the all-evidence map; this was not a pooled statistical sensitivity analysis.

### 3.5 Classification of reported factors associated with pain empathy

Reported factors were categorized separately for licensed clinical nurses and pre-licensure nursing students based on the research subjects. For clinical nurses, reported factors were grouped into six categories: ① Sociodemographic factors: gender (Dong et al., 2024), marital status (Dong et al., 2024; Li B. et al., 2025), religious belief (Dong et al., 2024; Shen et al., 2024), age (Dong et al., 2024; Li B. et al., 2025; Özbaş, 2025), educational attainment (Ren et al., 2023; Huang et al., 2025; Özbaş, 2025), parity (Sun et al., 2022; Zhang and Kuang, 2024; Li B. et al., 2025), only-child status (Huang et al., 2025); ② Occupational working factors: clinical department (Dong et al., 2024; Latimer et al., 2017; Jackson et al., 2017), professional title (Gao et al., 2022; Ren et al., 2023; Zhang and Kuang, 2024), working years (Wu et al., 2022; Sun et al., 2022), weekly working duration (Gao et al., 2022; Özbaş, 2025), time spent on pain evaluation (Gao et al., 2022), certification as pain resource nurse (Gao et al., 2022; Li B. et al., 2025), workplace violence exposure (Sun et al., 2022; Huang et al., 2025), professional quality of life (Sun et al., 2022), participation in hospital pain quality control (Wu et al., 2022), job satisfaction (Wu et al., 2022; Li B. et al., 2025; Huang et al., 2025; Özbaş, 2025); ③ Patient-related factor: visual visibility of patients’ painful symptoms (Paul-Savoie et al., 2018); ④ Intra-individual psychological and dispositional factors: psychological resilience (Dong et al., 2024; Huang et al., 2024), affective empathy (Gao et al., 2022; Zhang and Kuang, 2024), pain management self-efficacy (Luo et al., 2024), self-perceived pain tolerance (Wu et al., 2022; Li B. et al., 2025), personality characteristics (Wu et al., 2022; Huang et al., 2024), psychological wellbeing (Bourgault et al., 2015), coping styles (Huang et al., 2024; Li B. et al., 2025), transient emotional state (Wu et al., 2022; Wu et al., 2023), psychological toughness (Huang et al., 2025), knowledge sharing behavior (Ren et al., 2023), personal trauma and pain experience (Wu et al., 2022; Dong et al., 2024; Shen et al., 2024; Wu et al., 2023; Dobbs et al., 2014; Özbaş, 2025); ⑤ Social support factor: perceived social support (Zhang et al., 2025); ⑥ Educational and training factors: specialized pain empathy training (Wu et al., 2022; Dong et al., 2024; Sun et al., 2022; Wu et al., 2023; Özbaş, 2025), systematic pain knowledge training (Ren et al., 2023; Wu et al., 2023; Li B. et al., 2025; Özbaş, 2025), formal pain management training (Zhang and Kuang, 2024; Shen et al., 2024), narrative reading intervention (Kuo et al., 2022). For nursing students, reported factors were summarized into five categories: ① Sociodemographic factors: gender (Li et al., 2024), education level (Li et al., 2024), only-child status (Li et al., 2024); ② Academic and internship-related factors: academic grade (Culha, 2025), operating room internship experience (Li Q. et al., 2025), interpersonal relationships during clinical placement (Li Q. et al., 2025); ③ Individual psychological traits: preference for violent films (Li et al., 2024; Xiang et al., 2023), emotional intelligence (Culha, 2025), pain management self-efficacy (Culha, 2025), subjective pain tolerance (Xiang et al., 2023), cognitive and affective empathy (Xiang et al., 2023), sense of life meaning (Li Q. et al., 2025), pain-related fear (Yurdagül, 2025), personal pain experience (Xiang et al., 2023; Li B. et al., 2025; Culha, 2025); ④ Family-related support factor: parental rearing styles (Li et al., 2024; Li Q. et al., 2025); ⑤ Educational intervention factors: pain neurophysiology lectures (Mankelow et al., 2020), chronic pain immersive simulation training (Simko et al., 2021), humanistic nursing education (Li Q. et al., 2025), popularization of pain empathy-related theories (Xiang et al., 2023).

Observational correlates were distinguished from interventions or intervention-related exposures. Potential cohort overlap was assessed from authorship, sample size, population, setting, publication period, and reported outcomes. Definitive identity could not be established because recruitment dates, sites, and ethics identifiers were not consistently reported. To avoid double-counting, the three *n* = 1,601 reports were conservatively treated as one potentially overlapping cohort for evidence counts, and the two *n* = 27 reports were treated as a second potential cluster. Individual articles were retained because they addressed distinct analyses or outcomes, but findings within each cluster were not counted as independent replications. Supplementary Table S5 reports both publications and conservatively estimated independent cohorts.

## 4 Discussion

### 4.1 Overall level and reported factors associated with pain empathy

#### 4.1.1 Moderate overall level of pain empathy among nursing practitioners

Across the EPS studies, pain-empathy scores were most often reported at a moderate level, with two studies reporting high scores. Even in these two studies, the physical discomfort subdimension remained moderate. Heterogeneity in sampling regions, participants, and survey timing may account for the inconsistent findings. These results should be interpreted as a descriptive map rather than a pooled estimate. Multiple variables were reported in association with pain empathy, but their causal roles remain uncertain. The EPS-restricted descriptive comparison retained the same reported directions for education/training, resilience, pain knowledge, job satisfaction, and personal pain experience; no pooled cross-instrument estimate was calculated.

#### 4.1.2 Reported demographic associations

Demographic variables showed heterogeneous associations with pain empathy across nursing cohorts. Some studies reported higher scores among female nurses and those who were married, older, more highly educated, religious, had children, or were not only children. Explanations involving emotional sensitivity, family experience, clinical experience, or religious belief remain hypotheses because the included cross-sectional studies did not test these mechanisms (Yang et al., 2020; Li, 2022; Fabi and Leuthold, 2017).

For nursing students, gender, education level, and only-child status were reported as demographic correlates. Female students and those receiving bachelor-level education had higher scores in some studies. The reported association with only-child status does not establish a deficit in perspective-taking (Levett-Jones et al., 2019).

#### 4.1.3 Reported occupational and academic associations

Workplace and clinical characteristics were associated with differences in pain empathy. Staff in oncology and ICU settings, senior nurses, pain-resource nurses, and pain-management-team members had different scores in individual studies. Clinical exposure and training may help explain these patterns, but the cross-sectional evidence does not establish that they caused the observed differences (Li et al., 2022).

One cited study reported greater somatic and mental discomfort among nurses working over 50 h weekly. Workload and workplace violence were associated with less favorable empathy-related outcomes in the included studies, but causal direction was not established (Wang and Wang, 2020). Organizational support and prospective evaluation are therefore appropriate research and practice considerations.

For nursing undergraduates, academic progression, clinical placement, operating-room internship experience, and mentor relationships were associated with differences in empathy. These observations suggest possible educational influences but do not establish causality (Li B. et al., 2025).

#### 4.1.4 Training and intervention-related evidence

Professional training is a potentially modifiable area. Pain-knowledge training, empathy training, and narrative reading were reported as interventions, intervention-related exposures, or recommendations. Their effects should be distinguished from observational correlates and tested prospectively.

Student-oriented approaches included pain-neurophysiology education, chronic-pain simulation, humanistic nursing education, and introductory empathy courses. The quasi-experimental simulation study reported intervention-related change, whereas the remaining findings should not be interpreted as causal evidence.

#### 4.1.5 Visibility of clinical pain and possible Bias

One included study reported higher empathic responses to visible painful signs than to invisible chronic pain (Paul-Savoie et al., 2018). This single study suggests possible visibility bias, but its limited sample and design restrict confidence. Future studies could test structured assessment checklists and XR scenarios depicting invisible pain, with observer-rated decisions, behavioral responses, eye tracking, or physiological/neural measures used alongside self-report. Future work may also use predefined computational frameworks to integrate behavioral, eye-tracking, and physiological features, while validating algorithmic outputs against clinician-rated decisions.

#### 4.1.6 Psychological characteristics and reported associations

Psychological resilience, affective empathy, pain-management self-efficacy, perceived pain tolerance, personality, emotional state, knowledge sharing, and personal pain experience were reported as correlates of pain empathy. Proposed mechanisms involving emotional exhaustion, confidence, or personal pain thresholds were not tested by most included studies and require prospective confirmation (Luo et al., 2022).

Among nursing students, emotional intelligence, pain-management self-efficacy, violent-media exposure, and meaning in life were reported as correlates. The included studies do not establish that these characteristics caused changes in empathic responding (Miedzobrodzka et al., 2023).

Personal pain history showed mixed associations with pain empathy. Some reports described greater experiential insight, whereas others discussed possible empathic avoidance; causal direction remains uncertain (Jang et al., 2022).

#### 4.1.7 Social and familial support as reported correlates

Perceived social support was associated with sustained empathic engagement in one included study. Parental-rearing patterns were also reported as possible correlates of students’ empathy, but the evidence does not establish causal effects (Li Y. et al., 2025).

### 4.2 Limitations

This review is limited by the predominance of cross-sectional studies, heterogeneity between pain-specific and general-empathy measures, possible cohort overlap, inclusion of both nursing professionals and students, and an international evidence base that limits China-only conclusions. Cohort identity could not be definitively verified because recruitment sites, dates, and ethics identifiers were incompletely reported, so overlap clusters were counted conservatively. The search was restricted to Chinese- and English-language databases; the protocol was not prospectively registered, and grey literature was not searched. The item-level JBI appraisal and descriptive evidence-strength labels should not be interpreted as a pooled certainty-of-evidence assessment. Metric-specific estimates in Supplementary Tables S4, S5 were transcribed from the authors’ extraction table and were not converted or pooled across unlike metrics.

## 5 Conclusion

This scoping review mapped factors associated with pain empathy among nursing professionals and students. Reported factors included demographic, occupational, pain-related, psychological, educational, and social characteristics. Because most evidence was cross-sectional and the outcome constructs were heterogeneous, the findings describe associations rather than causal effects. Future studies should verify cohort independence and use longitudinal, randomized, and multimodal designs to evaluate pain-specific interventions across nursing education and practice.
