# Supplementary material for: A scoping review of factors associated with pain empathy among nursing professionals and students
Source: Front Psychol. 2026 Sep 7;17:1916376. doi: 10.3389/fpsyg.2026.1916376 (PMC13591469; doi:10.3389/fpsyg.2026.1916376)
Supplement: Supplementary file 1 [file Data_Sheet_1.pdf]

PRISMA-ScR Reporting Checklist

Section	Item	PRISMA-ScR checklist item	Location/status in revised manuscript
TITLE	1	Identify the report as a scoping review.	Title - Reported
ABSTRACT	2	Provide a structured summary including background, objectives, eligibility criteria, evidence sources, charting methods, results, and conclusions.	Abstract - Reported
INTRODUCTION	3	Describe the rationale for the review in the context of what is already known.	Introduction - Reported
INTRODUCTION	4	Provide an explicit statement of the questions and objectives being addressed with reference to population, concept, and context.	Introduction; Methods 1.1 - Reported
METHODS	5	Indicate whether a protocol exists; state where it can be accessed and provide registration information if available.	Methods 1.7; Limitations - No prospective registration
METHODS	6	Specify characteristics of the sources of evidence used as eligibility criteria and provide a rationale.	Methods 1.3 - Reported
METHODS	7	Describe all information sources and the date of the most recent search.	Methods 1.2; Supplementary S1 - Reported
METHODS	8	Present the full electronic search strategy for at least one database, including limits, so that it can be repeated.	Supplementary S1; PRESS record S7 - Reported for all databases
METHODS	9	State the process for selecting sources of evidence included in the scoping review.	Methods 1.4 - Reported
METHODS	10	Describe the methods of charting data and processes for obtaining and confirming data.	Methods 1.4 - Reported
METHODS	11	List and define all variables for which data were sought and any assumptions or simplifications.	Methods 1.4 and 1.6 - Reported
METHODS	12	If critical appraisal was performed, provide the rationale and describe the methods used.	Methods 1.5; Supplementary S3 - Reported
METHODS	13	Describe the methods of handling and summarizing charted data.	Methods 1.6 - Reported
RESULTS	14	Give numbers screened, assessed for eligibility, and included, with reasons for exclusions at each stage, ideally using a flow diagram.	Results 2.1; Figure 1; Supplementary S2-S2a - Reported
RESULTS	15	For each source of evidence, present characteristics for which data were charted and provide citations.	Results 2.2; Table 1 - Reported
RESULTS	16	If critical appraisal was performed, present the appraisal results.	Results 2.3; Supplementary S3 - Reported
RESULTS	17	For each included source of evidence, present relevant charted data related to the review questions and objectives.	Table 1; Supplementary S3-S5 - Reported
RESULTS	18	Summarize or present the charting results as they relate to the review questions and objectives.	Results 2.4-2.5; Supplementary S4-S5 - Reported
DISCUSSION	19	Summarize the main results, including concepts, themes, evidence types, and relevance to key groups.	Discussion and Conclusion - Reported
DISCUSSION	20	Discuss the limitations of the scoping review process.	Discussion 3.2 - Reported
DISCUSSION	21	Provide a general interpretation of the results with respect to the objectives and potential implications or next steps.	Conclusion - Reported
FUNDING	22	Describe sources of funding for the included evidence and for the scoping review, and describe the role of funders.	Funding statement - Reported: this research received no external funding

Supplementary Materials

Supplementary Table S1. Database-specific search strategies

Database	Platform/interface	Exact search strategy	Coverage	Final search date	Records (n)
PubMed	PubMed.gov	(("pain empathy"[Title/Abstract] OR "empathy for pain"[Title/Abstract] OR "pain sympathy"[Title/Abstract] OR "sympathy for pain"[Title/Abstract] OR "pain compassion"[Title/Abstract] OR "compassion for pain"[Title/Abstract]) AND ("Nurses"[MeSH Terms] OR "Nursing Staff"[MeSH Terms] OR "Students, Nursing"[MeSH Terms] OR nurse*[Title/Abstract] OR "nursing personnel"[Title/Abstract] OR "nursing staff"[Title/Abstract] OR "nursing student"[Title/Abstract] OR "nursing students"[Title/Abstract] OR "nursing intern"[Title/Abstract] OR "nursing interns"[Title/Abstract] OR "nursing assistant"[Title/Abstract] OR "nursing assistants"[Title/Abstract]))	Inception to 16 December 2025	16 December 2025	171
Embase	Embase.com	(('pain empathy':ti,ab,kw OR 'empathy for pain':ti,ab,kw OR 'pain sympathy':ti,ab,kw OR 'sympathy for pain':ti,ab,kw OR 'pain compassion':ti,ab,kw OR 'compassion for pain':ti,ab,kw) AND ('nurse'/exp OR 'nursing staff'/exp OR 'nursing student'/exp OR nurse*:ti,ab,kw OR 'nursing personnel':ti,ab,kw OR 'nursing staff':ti,ab,kw OR 'nursing student*':ti,ab,kw OR 'nursing intern*':ti,ab,kw OR 'nursing assistant*':ti,ab,kw))	Inception to 16 December 2025	16 December 2025	289
Web of Science Core Collection	Clarivate Web of Science Core Collection	TS=(("pain empathy" OR "empathy for pain" OR "pain sympathy" OR "sympathy for pain" OR "pain compassion" OR "compassion for pain") AND (nurse* OR "nursing personnel" OR "nursing staff" OR "nursing student*" OR "nursing intern*" OR "nursing assistant*"))	Inception to 16 December 2025	16 December 2025	644
Cochrane Library	Wiley Cochrane Library	((MeSH descriptor: [Nurses] explode all trees OR MeSH descriptor: [Nursing Staff] explode all trees OR MeSH descriptor: [Students, Nursing] explode all trees OR nurse*:ti,ab,kw OR "nursing personnel":ti,ab,kw OR "nursing staff":ti,ab,kw OR "nursing student*":ti,ab,kw OR "nursing intern*":ti,ab,kw OR "nursing assistant*":ti,ab,kw) AND ("pain empathy":ti,ab,kw OR "empathy for pain":ti,ab,kw OR "pain sympathy":ti,ab,kw OR "sympathy for pain":ti,ab,kw OR "pain compassion":ti,ab,kw OR "compassion for pain":ti,ab,kw))	Inception to 16 December 2025	16 December 2025	184
CNKI	China National Knowledge Infrastructure (CNKI)	SU=('疼痛共情'+'痛苦共情'+'疼痛同理心'+'疼痛同情'+'pain empathy'+'empathy for pain') AND SU=('护士'+'护理人员'+'护理学生'+'护生'+'护理实习生'+'护理辅助人员')	Inception to 16 December 2025	16 December 2025	33
VIP	VIP Chinese Science and Technology Journal Database	M=('疼痛共情' OR '痛苦共情' OR '疼痛同理心' OR '疼痛同情' OR 'pain empathy' OR 'empathy for pain') AND M=('护士' OR '护理人员' OR '护理学生' OR '护生' OR '护理实习生' OR '护理辅助人员')	Inception to 16 December 2025	16 December 2025	21
Wanfang Data	Wanfang Data Knowledge Service Platform	主题:('疼痛共情' OR '痛苦共情' OR '疼痛同理心' OR '疼痛同情' OR 'pain empathy' OR 'empathy for pain') AND 主题:('护士' OR '护理人员' OR '护理学生' OR '护生' OR '护理实习生' OR '护理辅助人员')	Inception to 16 December 2025	16 December 2025	1388
Chinese Biomedical Literature Database (CBM)	Chinese Biomedical Literature Service System (SinoMed/CBM)	('疼痛共情'[常用字段] OR '痛苦共情'[常用字段] OR '疼痛同理心'[常用字段] OR '疼痛同情'[常用字段] OR 'pain empathy'[常用字段] OR 'empathy for pain'[常用字段]) AND ('护理人员'[主题词] OR '学生,护理'[主题词] OR '护士'[常用字段] OR '护理人员'[常用字段] OR '护理学生'[常用字段] OR '护生'[常用字段] OR '护理实习生'[常用字段] OR '护理辅助人员'[常用字段])	Inception to 16 December 2025	16 December 2025	10

Supplementary Table S2. Full-text records excluded with reasons (n=55)

ID	Title	Year	Unique identifier	Exclusion category	Primary reason
P01	Physicians down-regulate their pain empathy response: an event-related brain potential study	2010	PMID 20080194	Ineligible population	The participants were physicians and matched controls, not nurses, nursing interns, nursing assistants, or nursing students.
P02	Observing physicians acting with different levels of empathy modulates later assessed pain tolerance	2022	PMID 34374180	Ineligible population	The participants were 60 women who viewed physician-scenario videos, and the outcome was the participants' pain tolerance rather than pain empathy in a nursing population.
P03	Patient-rated physician empathy and patient satisfaction during pain clinic consultations	2019	PMID 30919198	Ineligible population	Patients in a pain clinic rated physician empathy; the clinicians evaluated were physicians and the respondents were patients, neither of which was the target nursing population.
P04	Sharing pain and relief: neural correlates of physicians during treatment of patients	2014	PMID 23358155	Ineligible population	The study examined physicians' neural activity while treating patients; the participants were physicians rather than nursing personnel.
P05	Accountability and empathy effects on medical students' clinical judgments in a disability determination context for low back pain	2014	PMID 24952111	Ineligible population	The participants were medical students, not nursing students or nursing personnel.
P06	Medical undergraduate students' beliefs and attitudes toward pain: how do they mature?	2007	PMID 17258482	Ineligible population	The participants were medical undergraduates, whose professional background did not meet the nursing-population criterion in the PCC framework.
P07	Third-Year Medical Students' Reactions to Surgical Patients in Pain: Doubt, Distress, and Depersonalization	2018	PMID 30144537	Ineligible population	The participants were third-year medical students, not nursing students or nurses.
P08	Empathy and Osteopathic Manipulative Medicine: Is It All in the Hands?	2018	PMID 30178047	Ineligible population	The participants were osteopathic medical students rather than a nursing population.
P09	Verbal and non-verbal communication skills including empathy during history taking of undergraduate medical students	2018	PMID 29970069	Ineligible population	The participants were medical undergraduates, and the study focused on communication during history taking rather than the target nursing population.
P10	Desensitized gamers? Violent video game exposure and empathy for pain in adolescents - an ERP study	2023	PMID 37990996	Ineligible population	The participants were male adolescents aged 12-16 years, not nursing personnel or nursing students.
P11	Comorbid Chronic Pain and Depression: Patient Perspectives on Empathy	2016	PMID 27769812	Ineligible population	The participants were patients with comorbid chronic pain and depression, and the study examined their experience of clinicians' empathy rather than nurses' own pain empathy.
P12	Perceptions of the Provision of Pain Care Services from the Perspective of People with Parkinson's Disease: A Qualitative Descriptive Study	2025	PMID 40800566; PMCID PMC12341551	Ineligible population	The participants were people with Parkinson's disease, and the outcome was their perception of pain-care services rather than factors associated with pain empathy in a nursing population.

ID	Title	Year	Unique identifier	Exclusion category	Primary reason
S01	Empathy training in healthcare: an umbrella review	2025	PMID 41286773; PMCID PMC12751220	Ineligible study design	This was an umbrella review, a secondary study rather than an original quantitative, qualitative, or mixed-methods study.
S02	Measurement of empathy in nursing research: systematic review	2009	PMID 19146512	Ineligible study design	This was a systematic review rather than an original study.
S03	A systematic review of the effectiveness of empathy education for undergraduate nursing students	2019	PMID 30739841	Ineligible study design	This was a systematic review rather than an original intervention study.
S04	Distance-Based Educational Interventions for Empathy and Compassionate Care: A Systematic Review of International Applications	2025	PMCID PMC12576921	Ineligible study design	This was a systematic review rather than an original study.
S05	Evaluation of empathy measurement tools in nursing: systematic review	2009	PMID 19694842	Ineligible study design	This was a systematic review of measurement instruments rather than an original empirical study.
S06	Factors influencing empathy level among healthcare professionals: A systematic review and meta-analysis	2025	PMCID PMC12520288	Ineligible study design	This was a systematic review and meta-analysis rather than an original study.
S07	A systematic review of simulation to promote empathy in nursing education	2024	PMID 38265060	Ineligible study design	This was a systematic review rather than an original simulation-education study.
S08	Balint groups to improve empathy among medical professionals: a systematic review and meta-analysis	2024	PMID 39363356	Ineligible study design	This was a systematic review and meta-analysis rather than an original study.
S09	Empathy in Nursing Students: A Scoping Review	2023	PMID 37039440	Ineligible study design	This was a scoping review rather than an original study.
S10	A review of empathy education in nursing	2010	PMID 20137032	Ineligible study design	This was a narrative/literature review rather than an original study.
S11	Empathy-Based Education in Undergraduate Nursing: A Scoping Review	2024	PMID 38900258	Ineligible study design	This was a scoping review rather than an original educational intervention study.
S12	Artificial intelligence technologies and compassion in healthcare: A systematic scoping review	2023	PMID 36733854	Ineligible study design	This was a systematic scoping review rather than an original study.
S13	A historical analysis of the concept of empathy	1995	PMID 7499122	Ineligible study design	This was a historical concept analysis rather than an original empirical study.
S14	Clarification of the conceptualizations of empathy	2001	PMID 11489011	Ineligible study design	This was a conceptual clarification article rather than an original empirical study.
S15	Exploring empathy: a conceptual fit for nursing practice?	1992	PMID 1452181	Ineligible study design	This was a conceptual paper rather than an original study.
S16	On the nature and place of empathy in clinical nursing practice	1990	PMID 2198307	Ineligible study design	This was a theoretical/conceptual article rather than an original study.
S17	Exploring the Facets of Empathy and Pain in Clinical Practice: A Review	2017	PMID 28160400	Ineligible study design	This was a review article rather than an original study.
S18	The silent strain beyond the screen: conceptualizing digital compassion fatigue in nursing	2025	PMID 41310628; PMCID PMC12676866	Ineligible study design	This was a concept analysis using the Walker and Avant method rather than an original empirical study.
S19	Invisible nursing care: a concept analysis	2025	PMCID PMC12649001	Ineligible study design	This was a concept analysis rather than an original study.
S20	The nurse-patient relationship: a concept analysis	2021	PMID 34879785	Ineligible study design	This was a concept analysis rather than an original study.
S21	Nurses' attitudes towards communication with patients: a hybrid concept analysis	2025	PMCID PMC12512926	Ineligible study design	This was a hybrid concept analysis rather than an original study of associated factors.
S22	Vicarious Resilience in Hospital Nurses: A Concept Analysis	2025	PMCID PMC12469785	Ineligible study design	This was a concept analysis rather than an original study.
S23	Nurses' knowledge and attitudes towards acute pain management: a systematic review and meta-analysis	2023	PMID 36635867	Ineligible study design	This was a systematic review and meta-analysis rather than an original study.
S24	Nurses' pain assessment in patients with dementia: a systematic literature review	2020	PMID 33334680	Ineligible study design	This was a systematic literature review rather than an original study.
S25	Student nurses' knowledge and attitudes towards pain management: a systematic review	2021	PMID 34653890	Ineligible study design	This was a systematic review rather than an original study.
S26	Does Education Improve Nurses' Knowledge and Practice Regarding Pain Management? An Integrative Review	2023	PMID 36608996	Ineligible study design	This was an integrative review rather than an original educational intervention study.
S27	Paediatric pain management: knowledge, attitudes, barriers and facilitators among nurses - an integrative review	2018	PMID 29956310	Ineligible study design	This was an integrative review rather than an original study.
O01	Critical care nurses' perceived barriers and enablers of pain assessment and management	2023	PMID 36515086	Ineligible outcome or concept	The outcomes were barriers to and enablers of pain assessment and management; nurses' pain empathy was not the outcome being explained.
O02	Nurses' Knowledge, Attitudes, and Barriers Toward Pain Assessment and Management in Assir Region, Saudi Arabia	2023	PMID 35084247	Ineligible outcome or concept	The study measured knowledge, attitudes, and barriers related to pain assessment and management rather than pain empathy or its associated factors.
O03	Barriers and enablers of intensive care unit nurses' assessment and management of patients' pain	2021	PMID 33797160	Ineligible outcome or concept	The outcome was ICU nurses' pain-assessment and pain-management practice; pain empathy was not measured.
O04	Nurses' perception of pediatric pain and pain assessment in the Japanese PICU	2023	PMID 36762898	Ineligible outcome or concept	The study examined perceptions and assessment of pediatric pain rather than factors associated with nurses' pain empathy.
O05	Nurses' Knowledge and Attitude toward Pediatric Pain Management: A Cross-Sectional Study	2019	PMID 30528361	Ineligible outcome or concept	The outcomes were knowledge of and attitudes toward pediatric pain management; pain empathy was not measured.
O06	Complexity of Pain, Nurses' Knowledge, and Treatment Options	2015	PMID 26465627	Ineligible outcome or concept	The study focused on pain knowledge and treatment choices rather than pain-empathy level or associated factors.
O07	Pain Management Competency and its Associated Factors among Nurses	2023	PMID 36974848	Ineligible outcome or concept	The primary outcome was pain-management competency rather than pain empathy.
O08	Examining nurse empathy for infant procedural pain: Testing a new video measure	2011	PMID 22059191; PMCID PMC3202373	Ineligible outcome or concept	The study aimed to validate a video-based measure of empathy for infant procedural pain rather than identify factors associated with nurses' pain empathy.
O09	From knowledge to practice, intensive care nurses' pain management behaviors in Turkey: a cross sectional study	2025	PMID 41361305; PMCID PMC12683893	Ineligible outcome or concept	The primary outcome was ICU nurses' pain-management behavior; pain empathy was not treated as the principal dependent variable.
D01	Quality of Professional Life and Empathy of Healthcare Workers in an Emergency Department of General Hospital: A Cross-Sectional Study	2025	PMID 41404414; PMCID PMC12704177	Insufficient or non-extractable data	The mixed sample comprised 16 physicians, 39 nurses, and 15 nursing assistants; the available report presented combined JSE and regression results without a separately extractable estimate for pain-empathy factors in nurses.

ID	Title	Year	Unique identifier	Exclusion category	Primary reason
D02	Empathy levels among health professional students: a cross-sectional study at two universities in Australia	2014	PMID 24833947; PMCID PMC4014368	Insufficient or non-extractable data	The sample combined students from eight health professions; no separately extractable estimate of nursing students' pain-context empathy or associated factors was reported.
D03	Eyes on Newborns: How NICU Staff's Attention and Emotions Shape Neonatal Pain Assessment	2025	PMID 39899268; PMCID PMC11789839	Insufficient or non-extractable data	The population was reported as NICU staff/health personnel, and no separately extractable estimate of pain-empathy factors for nurses was available.
D04	Study on medical professionals' attitudes and awareness of multidisciplinary rehabilitation in pain syndromes	2025	PMID 40699082	Insufficient or non-extractable data	The study included mixed medical professionals, and the available record did not permit extraction of a nursing subgroup or a nursing pain-empathy outcome.
D05	The effect of knowledge brokering on nurses' empathy with patients receiving cardiac care: a study protocol	2020	PMID 32703257; PMCID PMC7376315	Insufficient or non-extractable data	This was a study protocol with no completed outcome data from which an effect direction or associated factor could be extracted.
D06	Current status and factors influencing compassion fatigue in clinical placement nursing students: a mixed-methods study protocol	2025	PMID 40983583; PMCID PMC12458761	Insufficient or non-extractable data	This was a pre-results study protocol with no extractable findings.
D07	A cluster randomized controlled trial for the Evaluation of routinely Measured PATient reported outcomes in HemodialYsis care (EMPATHY): a study protocol	2020	PMID 32778102; PMCID PMC7418420	Insufficient or non-extractable data	This was a trial protocol, and EMPATHY was an acronym for the project; no outcome data on nurses' pain empathy were available for extraction.

Supplementary Table S2a. Reports not retrieved (n=5)

ID	Title	Year	Unique identifier	Retrieval status	Reason
F01	Nursing students' assessment of pain and decision of triage for different ethnic groups: An experimental study	2015	PMID 25934558; DOI 10.1016/j.nedt.2015.04.004	Report not retrieved	Only a subscription DOI was located; no PMC or open-access full-text source was available, and the abstract was insufficient for full-text eligibility assessment.
F02	The Prediction of Nursing Students' Pediatric Pain Awareness Based on Levels of Child Liking and Basic Empathy	2025	PMID 40202212; DOI 10.1111/jcap.70013	Report not retrieved	Only the PubMed record and a subscription DOI were located; no open-access full text was available for eligibility assessment.
F03	Fostering Empathy and Motivation in Nursing Assistants Through Board Game-Based Learning: A Quasi-Experimental Study	2025	PMID 41199392; DOI 10.1111/scs.70137	Report not retrieved	Only a subscription DOI was located; no PMC or open-access full text was available to confirm whether the construct was pain specific.
F04	Factors Influencing Pain Management Practices Among Nurses in University Hospitals in Western Japan: A Cross-Sectional Study Using Hierarchical Multiple Regression Analysis	2025	PMID 40566658; DOI 10.1111/nhs.70143	Report not retrieved	Only a subscription DOI was located, with no PMC or open-access full text; full-text data were required to confirm the empathy measure, direction, and eligibility for this review.
F05	Empathy Affects Individualized Care Perception and Pain Knowledge in Nursing Students	2025 online/2026 issue	PMID 41402165; DOI 10.1016/j.pmn.2025.11.014	Report not retrieved	The article appeared online near the search cutoff, but no open-access full text was available to confirm its formal online date and whether the direction of the construct met the eligibility criteria.

Supplementary Table S3. Item-level JBI appraisal (n=27)

Study	Design	Q1	Q2	Q3	Q4	Q5	Q6	Q7	Q8	Q9	Q10
Dong2024	JBI analytical cross-sectional (8 items)	Y	Y	Y	Y	Y	Y	Y	Y	NA	NA
Gao2022	JBI analytical cross-sectional (8 items)	Y	Y	Y	Y	Y	Y	Y	Y	NA	NA
Ren2023	JBI analytical cross-sectional (8 items)	Y	Y	Y	Y	U	Y	Y	Y	NA	NA
Luo2024	JBI analytical cross-sectional (8 items)	Y	Y	U	Y	Y	Y	Y	Y	NA	NA
Sun2022	JBI analytical cross-sectional (8 items)	Y	Y	Y	Y	Y	Y	N	Y	NA	NA
Wu2022	JBI analytical cross-sectional (8 items)	Y	Y	Y	Y	U	Y	Y	Y	NA	NA
Zhang-H2024	JBI analytical cross-sectional (8 items)	Y	Y	Y	Y	Y	Y	Y	Y	NA	NA
Zhang-W2025	JBI analytical cross-sectional (8 items)	Y	Y	U	Y	Y	Y	Y	Y	NA	NA
Shen2024	JBI analytical cross-sectional (8 items)	Y	Y	Y	Y	U	Y	Y	Y	NA	NA
Li-Q2024	JBI analytical cross-sectional (8 items)	Y	Y	Y	Y	Y	Y	N	Y	NA	NA

Study	Design	Q1	Q2	Q3	Q4	Q5	Q6	Q7	Q8	Q9	Q10
Xiang2023	JBI analytical cross-sectional (8 items)	Y	Y	Y	Y	Y	Y	Y	Y	NA	NA
Guo2022	JBI qualitative (10 items)	Y	Y	U	Y	Y	U	Y	U	Y	Y
Huang-Y2024	JBI analytical cross-sectional (8 items)	Y	Y	Y	Y	Y	Y	Y	Y	NA	NA
Wu-L2023	JBI analytical cross-sectional (8 items)	Y	Y	Y	Y	Y	Y	Y	Y	NA	NA
Li-QX2025	JBI analytical cross-sectional (8 items)	Y	Y	Y	Y	U	Y	N	Y	NA	NA
Li-B2025	JBI analytical cross-sectional (8 items)	Y	Y	U	Y	Y	Y	Y	Y	NA	NA
Huang-YL2025	JBI analytical cross-sectional (8 items)	Y	Y	Y	Y	Y	Y	Y	Y	NA	NA
Bourgault2015	JBI analytical cross-sectional (8 items)	Y	Y	Y	Y	U	Y	Y	Y	NA	NA
Mankelow2021	JBI qualitative (10 items)	Y	Y	Y	Y	Y	Y	Y	U	Y	Y
Paul-Savoie2018	JBI analytical cross-sectional (8 items)	Y	Y	U	Y	Y	Y	N	Y	NA	NA
Latimer2017	JBI analytical cross-sectional (8 items)	Y	Y	Y	Y	U	Y	Y	Y	NA	NA
Jackson2017	JBI analytical cross-sectional (8 items)	Y	Y	Y	Y	Y	Y	Y	Y	NA	NA
Simko2021	JBI quasi-experimental (9 items)	Y	Y	Y	Y	Y	Y	Y	Y	Y	NA
Dobbs2014	JBI qualitative (10 items)	Y	Y	U	Y	Y	U	Y	U	Y	Y
Ozbas2025	JBI analytical cross-sectional (8 items)	Y	Y	Y	Y	Y	Y	N	Y	NA	NA
Yurdagul2025	JBI analytical cross-sectional (8 items)	Y	Y	Y	Y	Y	Y	Y	Y	NA	NA
Culha2025	JBI analytical cross-sectional (8 items)	Y	Y	Y	Y	U	Y	Y	Y	NA	NA

Note: Checklist type is shown for each study. Q1-Q10 denote consensus item positions; NA marks positions beyond the 8-item analytical cross-sectional or 9-item quasi-experimental checklist. Y=yes; N=no; U=unclear; NA=not applicable. No composite score was calculated. Item-level consensus ratings were derived from the two reviewers' assessments and adjudication record.

Supplementary Table S4. EPS-restricted descriptive comparison

Factor	All-evidence map	EPS supporting reports	EPS direction	Metric-specific estimate(s)	Interpretation
education/training	11 reports; positive	7 reports: Dong2024, Li-B2025, Li-QX2025, Ren2023, Shen2024, Sun2022, Wu-L2023	positive	r: 0.25 to 0.35; standardized β: 0.14 to 0.25	Direction retained in the EPS-restricted descriptive map
resilience	3 reports; positive	3 reports: Dong2024, Huang-Y2024, Huang-YL2025	positive	standardized β: 0.13 to 0.30	Direction retained in the EPS-restricted descriptive map
pain knowledge	2 reports; positive	1 reports: Wu-L2023	positive	r: 0.21	Direction retained in the EPS-restricted descriptive map
job satisfaction	4 reports; positive	3 reports: Huang-YL2025, Li-B2025, Wu2022	positive	r: 0.19; standardized β: 0.18 to 0.29	Direction retained in the EPS-restricted descriptive map
personal pain experience	9 reports; heterogeneous/mixed	6 reports: Dong2024, Li-QX2025, Shen2024, Wu-L2023, Wu2022, Xiang2023	heterogeneous/mixed	r: 0.20 to 0.33; standardized β: 0.16	Direction retained in the EPS-restricted descriptive map

Note: This is a construct-restricted descriptive comparison, not a pooled statistical sensitivity analysis. Unlike effect metrics are displayed separately and are not converted or pooled. Numeric estimates were transcribed from the authors' extraction table.

Supplementary Table S5. Factor evidence matrix

Category/factor	Supporting reports/design	Participant/sample characteristics	Direction	Metric-specific estimate(s)	Descriptive strength	Source of estimate
Demographic / age	Li-B2025, Ozbas2025, Ren2023; 3 reports/3 conservative cohorts; Cross-sectional study	Clinical nurses (n=9168); Clinical nurses (n=1601); Surgical/operating-room/ICU nurses (n=242)	heterogeneous/mixed	NR	limited	Direction recorded; quantitative estimate NR
Demographic / childbearing status	Sun2022, Zhang-H2024; 2 reports/2 conservative cohorts; Cross-sectional study	Pediatric nurses (n=132); Clinical nurses (n=254)	heterogeneous/mixed	NR	limited	Direction recorded; quantitative estimate NR
Demographic / education/academic level	Culha2025, Huang-YL2025, Li-Q2024, Ozbas2025, Ren2023; 5 reports/5 conservative cohorts; Cross-sectional study	Clinical nurses (n=9168); Nursing interns (n=230); Clinical nurses (n=1601); Surgical/operating-room/ICU nurses (n=242); Nursing students (n=326)	positive	r: 0.19 to 0.36; standardized β: 0.19 to 0.31	moderate	Authors' extraction table

Category/factor	Supporting reports/design	Participant/sample characteristics	Direction	Metric-specific estimate(s)	Descriptive strength	Source of estimate
Demographic / gender	Dong2024, Li-Q2024; 2 reports/2 conservative cohorts; Cross-sectional study	Clinical nurses (n=953); Nursing interns (n=230)	heterogeneous/mixed	NR	limited	Direction recorded; quantitative estimate NR
Demographic / marital status	Dong2024, Li-B2025; 2 reports/2 conservative cohorts; Cross-sectional study	Clinical nurses (n=953); Clinical nurses (n=1601)	heterogeneous/mixed	NR	limited	Direction recorded; quantitative estimate NR
Demographic / only-child status	Huang-YL2025, Li-Q2024; 2 reports/2 conservative cohorts; Cross-sectional study	Nursing interns (n=230); Clinical nurses (n=1601)	heterogeneous/mixed	NR	limited	Direction recorded; quantitative estimate NR
Demographic / religious belief	Dong2024, Shen2024; 2 reports/2 conservative cohorts; Cross-sectional study	Clinical nurses (n=953); Infectious-disease nurses (n=271)	heterogeneous/mixed	NR	limited	Direction recorded; quantitative estimate NR
Education/training / education/training	Dong2024, Guo2022, Li-B2025, Li-QX2025, Mankelow2021, Ozbas2025, Ren2023, Shen2024, Simko2021, Sun2022, Wu-L2023; 11 reports/11 conservative cohorts; Cross-sectional study; Qualitative research; Quasi-experimental study	Clinical nurses (n=953); Clinical nurses (n=9168); Pediatric nurses (n=132); Infectious-disease nurses (n=271); Urology nurses (n=9); Clinical nurses (n=177); Nursing students (n=423); Clinical nurses (n=1601); Nursing students (2 of 12 participants) (n=12); Nursing/interprofessional students (n=182); Surgical/operating-room/ICU nurses (n=242)	positive	Hedges g: 0.46; r: 0.19 to 0.35; standardized β: 0.14 to 0.25	moderate	Authors' extraction table
Education/training / empathy awareness	Wu2022, Xiang2023; 2 reports/2 conservative cohorts; Cross-sectional study	Clinical nurses (n=489); Vocational nursing students (n=653)	positive	r: 0.25; standardized β: 0.25	limited	Authors' extraction table
Education/training / pain knowledge	Ozbas2025, Wu-L2023; 2 reports/2 conservative cohorts; Cross-sectional study	Clinical nurses (n=177); Surgical/operating-room/ICU nurses (n=242)	positive	r: 0.21 to 0.38	limited	Authors' extraction table
Pain-related / fear of pain	Yurdagul2025; 1 reports/1 conservative cohorts; Cross-sectional study	Nursing students (n=396)	negative	r: -0.38	very limited	Authors' extraction table
Pain-related / pain visibility	Paul-Savoie2018; 1 reports/1 conservative cohorts; Cross-sectional study	Clinical nurses (n=21)	positive	r: 0.22	very limited	Authors' extraction table
Pain-related / perceived pain tolerance	Li-B2025, Wu2022, Xiang2023; 3 reports/3 conservative cohorts; Cross-sectional study	Clinical nurses (n=489); Vocational nursing students (n=653); Clinical nurses (n=1601)	negative	r: -0.34; standardized β: -0.25 to -0.16	limited	Authors' extraction table
Pain-related / personal pain experience	Culha2025, Dobbs2014, Dong2024, Li-QX2025, Ozbas2025, Shen2024, Wu-L2023, Wu2022, Xiang2023; 9 reports/9 conservative cohorts; Cross-sectional study; Qualitative research	Clinical nurses (n=953); Clinical nurses (n=489); Infectious-disease nurses (n=271); Vocational nursing students (n=653); Clinical nurses (n=177); Nursing students (n=423); Nurse auxiliaries (n=28); Surgical/operating-room/ICU nurses (n=242); Nursing students (n=326)	heterogeneous/mixed	r: 0.20 to 0.33; standardized β: 0.16	limited	Authors' extraction table
Psychological/emotional / coping style	Huang-Y2024, Li-B2025; 2 reports/1 conservative cohorts; Cross-sectional study	Clinical nurses (n=1601)	heterogeneous/mixed	NR	very limited	Direction recorded; quantitative estimate NR
Psychological/emotional / emotional state	Wu-L2023, Wu2022; 2 reports/2 conservative cohorts; Cross-sectional study	Clinical nurses (n=489); Clinical nurses (n=177)	negative	r: -0.39 to -0.21	limited	Authors' extraction table
Psychological/emotional / emotional/cognitive empathy	Culha2025, Gao2022, Xiang2023, Zhang-H2024; 4 reports/4 conservative cohorts; Cross-sectional study	Clinical nurses (n=687); Clinical nurses (n=254); Vocational nursing students (n=653); Nursing students (n=326)	positive	r: 0.20 to 0.30; standardized β: 0.29 to 0.30	moderate	Authors' extraction table
Psychological/emotional / meaning in life	Li-QX2025; 1 reports/1 conservative cohorts; Cross-sectional study	Nursing students (n=423)	positive	r: 0.24	very limited	Authors' extraction table
Psychological/emotional / pain-management self-efficacy	Culha2025, Luo2024; 2 reports/2 conservative cohorts; Cross-sectional study	ICU nurses (n=324); Nursing students (n=326)	positive	r: 0.19 to 0.21	limited	Authors' extraction table
Psychological/emotional / personality traits	Huang-YL2025, Wu2022; 2 reports/2 conservative cohorts; Cross-sectional study	Clinical nurses (n=489); Clinical nurses (n=1601)	heterogeneous/mixed	NR	limited	Direction recorded; quantitative estimate NR
Psychological/emotional / psychological/professional well-being	Bourgault2015, Sun2022; 2 reports/2 conservative cohorts; Cross-sectional study	Pediatric nurses (n=132); Emergency nurses (n=30)	positive	r: 0.22 to 0.30	limited	Authors' extraction table
Psychological/emotional / resilience	Dong2024, Huang-Y2024, Huang-YL2025; 3 reports/2 conservative cohorts; Cross-sectional study	Clinical nurses (n=953); Clinical nurses (n=1601)	positive	standardized β: 0.13 to 0.30	limited	Authors' extraction table
Social/environment / internship relationships	Li-QX2025; 1 reports/1 conservative cohorts; Cross-sectional study	Nursing students (n=423)	positive	r: 0.35	very limited	Authors' extraction table
Social/environment / parental/family rearing	Li-Q2024, Li-QX2025; 2 reports/2 conservative cohorts; Cross-sectional study	Nursing interns (n=230); Nursing students (n=423)	heterogeneous/mixed	NR	limited	Direction recorded; quantitative estimate NR
Social/environment / social support	Zhang-W2025; 1 reports/1 conservative cohorts; Cross-sectional study	ICU nurses (n=394)	positive	r: 0.35	very limited	Authors' extraction table
Work/learning / clinical department	Dong2024; 1 reports/1 conservative cohorts; Cross-sectional study	Clinical nurses (n=953)	heterogeneous/mixed	NR	very limited	Direction recorded; quantitative estimate NR
Work/learning / clinical placement	Li-QX2025; 1 reports/1 conservative cohorts; Cross-sectional study	Nursing students (n=423)	positive	r: 0.18	very limited	Authors' extraction table
Work/learning / clinical seniority/experience	Gao2022, Ozbas2025, Ren2023, Sun2022, Wu2022, Zhang-H2024; 6 reports/6 conservative cohorts; Cross-sectional study	Clinical nurses (n=687); Clinical nurses (n=9168); Pediatric nurses (n=132); Clinical nurses (n=489); Clinical nurses (n=254); Surgical/operating-room/ICU nurses (n=242)	positive	r: 0.25 to 0.39; standardized β: 0.17 to 0.26	moderate	Authors' extraction table
Work/learning / job satisfaction	Huang-YL2025, Li-B2025, Ozbas2025, Wu2022; 4 reports/3 conservative cohorts; Cross-sectional study	Clinical nurses (n=489); Clinical nurses (n=1601); Surgical/operating-room/ICU nurses (n=242)	positive	r: 0.19 to 0.24; standardized β: 0.18 to 0.29	limited	Authors' extraction table
Work/learning / knowledge sharing	Ren2023; 1 reports/1 conservative cohorts; Cross-sectional study	Clinical nurses (n=9168)	positive	standardized β: 0.18	very limited	Authors' extraction table
Work/learning / neural correlates	Jackson2017; 1 reports/1 conservative cohorts; Cross-sectional study	Neonatal/pediatric intensive-care nurses (n=27)	heterogeneous/mixed	NR	very limited	Direction recorded; quantitative estimate NR
Work/learning / occupational characteristics	Jackson2017, Latimer2017; 2 reports/1 conservative cohorts; Cross-sectional study	Neonatal/pediatric intensive-care nurses (n=27)	heterogeneous/mixed	NR	very limited	Direction recorded; quantitative estimate NR
Work/learning / pain-assessment time	Gao2022; 1 reports/1 conservative cohorts; Cross-sectional study	Clinical nurses (n=687)	positive	standardized β: 0.25	very limited	Authors' extraction table

Category/factor	Supporting reports/design	Participant/sample characteristics	Direction	Metric-specific estimate(s)	Descriptive strength	Source of estimate
Work/learning / pain-management role	Gao2022, Li-B2025, Wu2022, Zhang-H2024; 4 reports/4 conservative cohorts; Cross-sectional study	Clinical nurses (n=687); Clinical nurses (n=489); Clinical nurses (n=254); Clinical nurses (n=1601)	positive	r: 0.22 to 0.33; standardized β: 0.27 to 0.32	moderate	Authors' extraction table
Work/learning / shift	Ozbas2025; 1 reports/1 conservative cohorts; Cross-sectional study	Surgical/operating-room/ICU nurses (n=242)	heterogeneous/mixed	NR	very limited	Direction recorded; quantitative estimate NR
Work/learning / violent-media exposure/preference	Li-Q2024, Xiang2023; 2 reports/2 conservative cohorts; Cross-sectional study	Nursing interns (n=230); Vocational nursing students (n=653)	negative	r: -0.23; standardized β: -0.26	limited	Authors' extraction table
Work/learning / workload/shift	Gao2022; 1 reports/1 conservative cohorts; Cross-sectional study	Clinical nurses (n=687)	negative	standardized β: -0.23	very limited	Authors' extraction table
Work/learning / workplace violence	Huang-YL2025, Sun2022; 2 reports/2 conservative cohorts; Cross-sectional study	Pediatric nurses (n=132); Clinical nurses (n=1601)	negative	r: -0.37; standardized β: -0.22	limited	Authors' extraction table

Note: Descriptive evidence strength is not GRADE. Moderate = at least three conservatively estimated independent cohorts with a generally consistent direction and at least one quantitative estimate; limited = two cohorts or heterogeneous/incomplete estimates; very limited = one cohort or no extractable quantitative estimate. JBI findings were used to temper interpretation. Unlike metrics are shown separately and are not pooled.

Supplementary Table S6. Reviewer agreement

Stage	Items	Both yes	R1 yes/R2 no	R1 no/R2 yes	Both no	Observed agreement	Expected agreement	Kappa
Title/abstract screening	2197	82	17	17	2081	0.9845	0.9139	0.82
Retrieval/eligibility disposition (87 reports sought)	87	23	4	4	56	0.9080	0.5719	0.79
Data-extraction form completeness	27	14	1	1	11	0.9259	0.5062	0.85

Note: Cohen's kappa was calculated as kappa=(Po-Pe)/(1-Pe). At the 87-report retrieval/eligibility stage, five not-retrieved reports were retained in the agreement denominator as a separate negative disposition; they were not counted among the 55 full-text exclusions. After adjudication, the 87 reports comprised 27 included studies, 55 full-text exclusions, and 5 not retrieved.

Supplementary Table S7. PRESS 2015 review record

No.	PRESS element	Verification performed	Recorded result/action
1	Translation of the research question	Pain-empathy and nursing-population concepts represented in all database adaptations	Checked; no concept omitted
2	Boolean and proximity operators	AND/OR structure and database-specific proximity syntax	Checked and standardized
3	Subject headings	MeSH/Emtree or database-appropriate controlled vocabulary where supported	Checked against platform syntax
4	Text words	Synonyms, spelling variants, truncation, and field tags	Checked
5	Spelling, syntax, and line structure	Parentheses, quotation marks, field codes, and database translation	Checked
6	Limits and filters	Coverage from database inception to 16 December 2025; no undocumented filters	Checked
7	Overall peer-review disposition	Second reviewer completed the PRESS check before resubmission	No additional search-string change was required

Note: The PRESS check was completed by a second reviewer before resubmission; no additional search-string change was required.
